# Egg yolk-free cryopreservation of bull semen

**DOI:** 10.1371/journal.pone.0223977

**Published:** 2019-10-15

**Authors:** Muhammad Anzar, Kosala Rajapaksha, Lyle Boswall

**Affiliations:** 1 Agriculture and Agri-Food Canada, Saskatoon Research and Development Center, Saskatoon, Saskatchewan, Canada; 2 Department of Veterinary Biomedical Sciences, Western College of Veterinary Medicine, University of Saskatchewan, Saskatoon, Saskatchewan, Canada; University of Porto, PORTUGAL

## Abstract

Egg yolk is a common ingredient of mammalian semen extender to protect sperm against initial cold shock. However, egg yolk has biosecurity risks. Our main objectives were to cryopreserve bull semen without egg yolk using exogenous cholesterol and to study the protective role of glycerol in egg yolk-free semen extender. Other objectives were to compare protein profiles and in vitro fertilization potential of bull sperm frozen with and without egg yolk. In first experiment, semen was either diluted in conventional tris-egg yolk glycerol (TEYG control) extender or first treated with cholesterol-cyclodextrin complex (CC, 2 mg/ml semen) followed by dilution in egg yolk-free tris-glycerol (TG) extender (collectively called as “CC+TG”) at 22°C or 4°C, and frozen. Post-thaw sperm motion characteristics were similar between CC+TG and TEYG control extenders, and temperature of glycerol addition. In second experiment, semen was frozen in CC+TG extender varying in glycerol concentration (7 to 0%; v/v). Post-thaw sperm quality decreased with the decline in glycerol concentration in TG extender, even higher concentration of CC complex (3 or 4 mg/ml semen) could not protect sperm in the absence of glycerol in TG extender. In third experiment, SDS electrophoresis of proteins from fresh sperm and sperm frozen in CC+TG, and TEYG control extenders was conducted. Protein profiles in fresh sperm and CC+TG frozen sperm were almost similar. Egg yolk proteins bound tightly with sperm plasma membrane. In fourth experiment, in vitro fertilization potentials of sperm frozen in TEYG control and CC+TG extenders were tested. Cleavage and blastocyst rates of semen frozen in CC+TG and TEYG control extenders were similar. In conclusion, cholesterol-cyclodextrin replaced egg yolk from the semen extender; glycerol remained essential for egg yolk-free sperm cryopreservation; and CC+TG extender did not modify sperm plasma membrane CC+TG whereas egg yolk extender changed the plasma membrane composition of bull sperm.

## Introduction

Cryopreservation of semen is widely used for conservation of animal genetic resources and exploitation of genetically superior sires through artificial insemination. Mammalian sperm undergo thermal shocks during initial cooling from room to refrigerator temperature (supra-zero phase) and then during deep freezing (sub-zero phase). Since the discovery of egg yolk as an ingredient of bull semen extender [1], it has been extensively used in mammalian semen cryopreservation to protect sperm against initial cold shock [2,3]. The exact mechanism of sperm protection by egg yolk during initial cooling phase is not fully known. Upon ejaculation, binder of sperm proteins (BSPs) present in seminal plasma cause efflux of phospholipids and cholesterol from plasma membrane. Low density lipoproteins (LDLs) in egg yolk protect sperm by sequestering BSPs in seminal plasma [4,5]. Egg yolk LDLs bind with sperm plasma membrane following dilution with egg yolk extender [6]. Alternately, milk-based extenders are used for bull semen throughout the world. Lipoproteins in homogenized milk and casein micelles in skimmed milk protect sperm during initial cooling phase and storage at refrigerated temperature [7]. Unfortunately, egg yolk and milk extenders are difficult to standardize because they vary from producer to producer and introduce microbial contamination [8,9]. The artificial insemination industry has been facing biosecurity risks through the use of animal products (egg yolk and milk) in semen cryopreservation procedure [10]. The Canadian Food Inspection Agency and World Organization for Animal Health recommended that egg yolk or milk used in semen extender should be free of pathogens or sterilized [11,12]. Therefore, there is a dire need of elimination of animal products from cryopreservation procedure to comply with international regulations on import and export. Several research efforts have been made to replace egg yolk and milk in extender. Soy lecithin from soy bean has been used to replace egg yolk in extenders. Soy bean-based extenders are commercially available for cryopreservation of bull semen. However, soy bean extender varies from batch to batch, and yields inconsistent fertility rates [9,13]. Recently, liposomes made up of pure phospholipids have been introduced for cryopreservation of bovine semen [14,15]. However, pure phospholipids are quite expensive in the market.

Cholesterol, an integral part of the sperm plasma membrane, strengthens and protects membrane structures even below phase transition temperature by resisting change in the structural composition of phospholipids’ hydrocarbon chains [16]. Cholesterol content in sperm plasma membrane determines their cryotolerance. Rabbit and human sperm, with high cholesterol:phospholipid (C:P) molar ratios i.e. 0.88 and 0.99 respectively, are highly resistant to cryopreservation in comparison to boar, stallion and bull in which C:P molar ratios are very low i.e. 0.26, 0.36 and 0.45, respectively [17–20]. Bull sperm have better cryosurvival compared to boar and stallion semen. During cryopreservation, cholesterol content of sperm plasma membrane is removed which causes their premature capacitation [21]. Cholesterol (hydrophobic) can be removed from or incorporated in sperm plasma membranes using cyclodextrins [22]. Cyclodextrins are oligosaccharides having hydrophobic interior core and hydrophilic exterior. They bind with exogenous cholesterol, form soluble complexes and deliver cholesterol to cell membranes [23]. Treatment of sperm with methyl-β-cyclodextrin preloaded with cholesterol before dilution in egg yolk extender improved post-thaw survival in bull [24], stallion [25] and pig [26] semen. Recently, 2-hydroxypropyl-beta-cyclodextrin cholesterol (HPβCD-C) complex replaced egg yolk from extender for cryopreservation of equine sperm [27]. In this study, it was hypothesized that exogenous cholesterol-cyclodextrin (CC) complex can strengthen bull sperm plasma membrane sufficient enough to exclude egg yolk or milk from a conventional extender.

Glycerol, the most common permeating cryoprotectant for mammalian semen cryopreservation, protects sperm from thermal shock below freezing point. It provides a ‘salt-buffering’ mechanism and reduces freezing stress [28], binds with metallic ions [29], dehydrates cells [30] and reduces total ice volume during water solidification and thus prevents fracture in frozen solutions [31]. In bovine, the post-thaw sperm motility and fertility depends upon the concentrations of glycerol in extender [32]. Glycerol and egg yolk have a synergistic effect in protecting bull sperm during cryopreservation [2]. In equine semen cryopreservation, the glycerol concentration was reduced from 3% to 1% after treating sperm with HPβCD-C complex [27]. Therefore, it was hypothesized that CC treatment will reduce the glycerol concentration required for bull semen cryopreservation also. In this study, the appropriate concentration of glycerol in egg yolk-free tris-glycerol (TG) extender for cryopreservation of bull semen was also investigated.

Egg yolk proteins bind with sperm plasma membrane [6]; therefore, we hypothesized that egg yolk proteins mask the innate proteins of sperm plasma membrane whereas treatment of sperm with CC will not cause major changes in sperm membrane. In addition to hygienic benefits, the dilution of semen with protein-free extender would provide an opportunity to study mammalian sperm proteomics following cryopreservation.

The main goal of this study was to cryopreserve bull semen without adding any exogenous protein in extender. The specific objectives were to compare the post-thaw quality of bull sperm pre-exposed to exogenous cholesterol-cyclodextrin complex and frozen in egg yolk-free tris-glycerol (CC+TG) extender with conventional tris-egg yolk-glycerol (TEYG control) extender, to investigate the role of glycerol in egg yolk-free semen cryopreservation, to determine protein profiles of bull sperm frozen in CC+TG and TEYG control extender and to assess *in vitro* fertilizing ability of sperm frozen in CC+TG or TEYG control extenders.

## Materials and methods

### Preparation of extenders

Tris-citric acid (TCA) extender was prepared by dissolving tris 3.03% (w/v), citric acid monohydrate 1.74% (w/v) and fructose 1.24% (w/v) in Milli-Q distilled water. Tris-citric acid-egg yolk-glycerol (TEYG control) extender contained egg yolk 20% (v/v) and glycerol 7% (v/v) in TE. Tris-citric acid-glycerol (TG; egg yolk-free) extender contained glycerol 14, 10, 6, 2 and 0% (v/v) in TCA extender, depending upon experiment. All TCA, TEYG control and TG extenders contained gentamycin (500 μg/ml), tylan (100 μl/ml) and linco-spectin (300/600 μg/ml), and were centrifuged at 12000x g for 15 min at 4°C. The supernatant was stored at -20°C until the day of experiment. In addition, TEYG and TG extenders of double concentration (2X) were also prepared for the first experiment.

### Preparation of cholesterol-cyclodextrin complex (CC)

Cholesterol (Sigma-Aldrich, Oakville, ON) and methyl β-cyclodextrin (Sigma-Aldrich, Oakville, ON) complex was prepared as described earlier [24]. Solution A was prepared by dissolving cholesterol 200 mg in 1 ml chloroform. Solution ‘B’ was prepared by dissolving 1 g methyl β-cyclodextrin in 2 ml methanol. An aliquot solution A (0.45 ml) was added to solution B and stirred well until the solution became homogenous. The cholesterol-cyclodextrin complex (CC) was slowly dried with nitrogen gas in a glass petri dish, kept overnight in a desiccator, and stored at 22°C in a glass container. On the day of experiment, a working solution was prepared by dissolving CC in TCA (50 mg/ml) at 37°C and vortexing gently.

### Semen collection and initial evaluation

Four beef bulls were used in this study. These bulls were stationed at Goodale Research Farm, University of Saskatchewan, Saskatoon (52° 08’N, 106° 37’W). Semen was collected from each bull, once a week (Monday), via electroejaculation procedure approved by the Animal Care Committee, University of Saskatchewan, Saskatoon (Animal Use Protocol # 20100150). Semen was transported to the Cryobiology Laboratory at the University of Saskatchewan, in a mobile incubator (37°C) within 1 h. Semen ejaculates were initially evaluated for sperm motility and concentration with a computer-assisted sperm analyzer (CASA; SpermVision 3.5, Minitube Canada, Ingersoll, Ontario) [33], as mentioned below.

### Cryopreservation of semen without egg yolk (exogenous protein)

**Fig 1:**
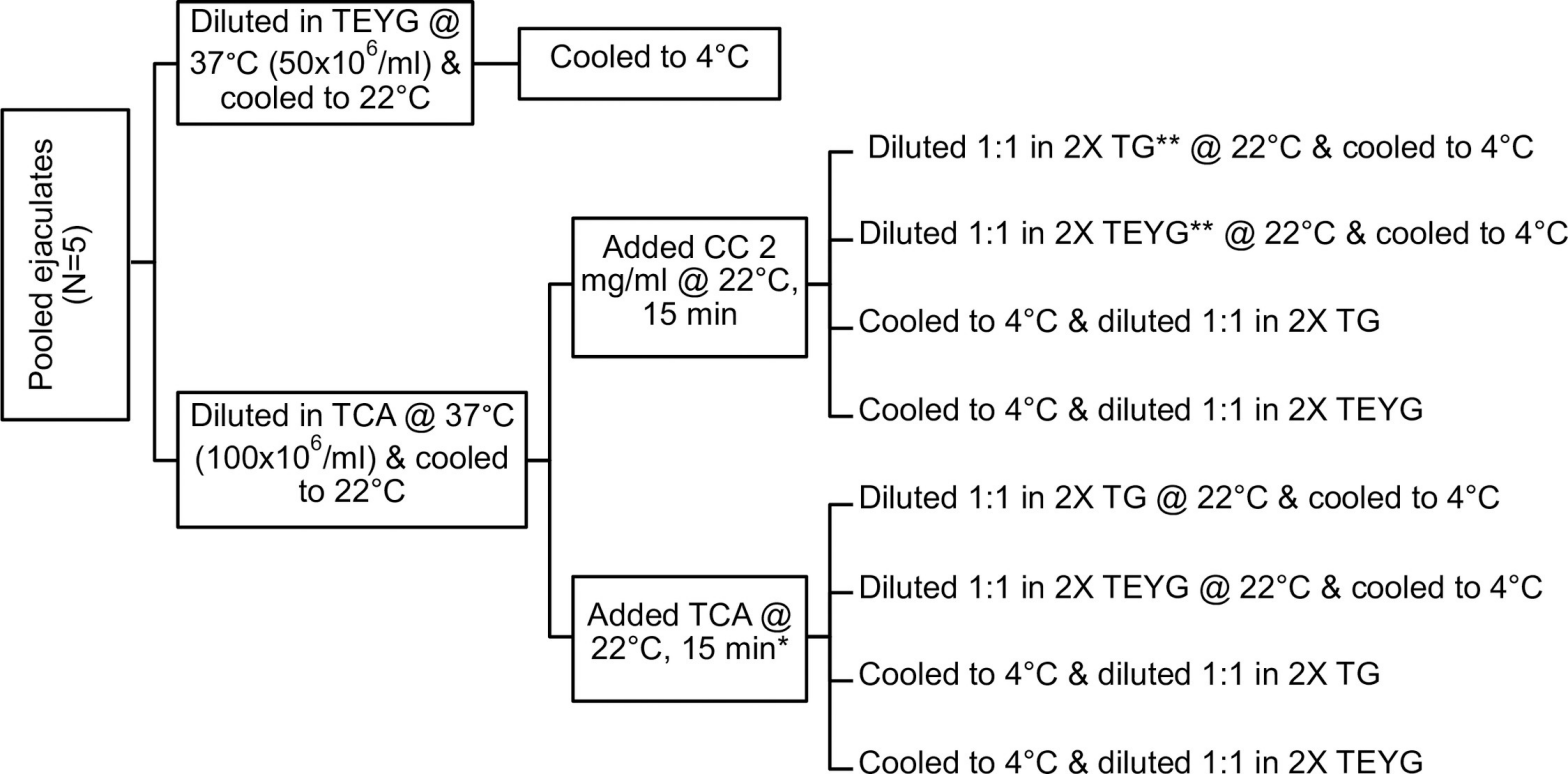
Experimental design of semen processing with and without egg yolk. Control and treatment semen samples were frozen using programmable freezer, as mentioned in materials and methods section. *At 22°C, TCA volume equivalent to the volume of CC treatment was added. **After treatment with CC or TE, semen was diluted 1:1 (v/v) with 2X TG or 2X TEYG extender either at 22°C or 4°C to final sperm concentration 50x10^6^/ml. CC: cholesterol-cyclodextrin complex; TE: tris-citric acid extender; TEYG: tris-citric acid-egg yolk-glycerol extender; TG: tris-citric acid-glycerol extender.

This experiment was designed to assess if treatment of bull sperm with CC complex can eliminate egg yolk from semen extender and to determine the protective role of CC during initial cooling phase. An outline of this experiment is presented in Fig 1. After initial evaluation, semen ejaculates, possessing sperm motility >60% and concentration >400x10^6^/ml, were pooled to eliminate bull effect. A small portion of the pooled semen was diluted to 50x10^6^ sperm/ml in conventional TEYG extender (called “TEYG control semen”) at 37°C and frozen as mentioned below. A major portion of semen was first diluted to 100x10^6^ sperm/ml with TCA extender at 37°C and cooled to 22°C in a water bath. At 22°C, semen was treated with either CC (2 mg/ml semen) or equal volume of plain TCA extender, for 15 min. One set of CC- and TCA-treated semen samples was diluted 1:1 (v/v) with 2X TEYG or 2X TG extenders (final sperm concentration 50x10^6^/ml) at 22°C and slowly cooled to 4°C in 90 min. Another set of CC- and TCA-treated semen samples was first slowly cooled to 4°C in 90 min, and then diluted 1:1 (v/v) with 2X TEYG or 2X TG extenders (final sperm concentration 50x10^6^/ml) at 4°C. Sperm treated with CC and diluted in TG extender (containing 7% glycerol) were collectively called “CC2+TG7% semen”. For freezing, both control and CC2+TG7% semen samples were slowly cooled from 22°C to 4°C in a walk-in cold room (4°C) in 90–100 min. Semen was packaged in 0.5 ml French straws and frozen to -80°C in a programmable cell freezer (ICE-CUBE 14-S, Sy-Lab Version 1.30, Gerate GmbH, Neupurkdersdof, Austria) using a freezing curve, as reported earlier [33]. Briefly, semen straws were cooled from +4°C to -10°C @ -3°C/min, and from -10°C to -80°C @ -40°C/min. At -80°C, semen straws were plunged in liquid nitrogen and stored for at least 24 h. Three semen straws per treatment per replicate were thawed in a water bath at 37°C for 60 s, and semen was pooled. Post-thaw motility characteristics were evaluated using CASA, as described below. This experiment was replicated on five different pooled ejaculates collected from four bulls.

### Effect of cholesterol-cyclodextrin complex and glycerol combinations in extender on cryopreservation of semen without egg yolk

This experiment was conducted to determine the need of glycerol in egg yolk-free cryopreservation of bull semen. Control semen containing 20% (v/v) egg yolk and 7% (v/v) glycerol was processed as in previous experiment. For treatment groups, semen was initially diluted with TCA extender to 100x10^6^ sperm/ml. Based on results from the first experiment, sperm were treated with CC (2 mg/ml semen) at 22°C for 15 min and then diluted 1:1 (v/v) with TG extender at 22°C such that the final glycerol concentration was 7, 5, 3, 1 or 1% (v/v). In addition, bull sperm were treated with higher concentrations of CC (3 or 4 mg/ml semen) and diluted with plain TCA (without glycerol) to determine if CC can replace glycerol also. These treatment groups were designated as CC2+TG7%, CC2+TG5%, CC2+TG3%, CC2+TG1%, CC2+TG0%, CC3+TG0% and CC4+TG0% in subsequent text.

Semen diluted and frozen with TEYG control was considered as the first control and semen frozen with CC2+TG7% was considered as a second control. All semen samples were cooled to 4°C and frozen in programmable cell freezer as described in previous experiment. Three semen straws per treatment per replicate were thawed in a water bath at 37°C for 60 s, and semen was pooled. Post-thaw motility and structural characteristics were evaluated using CASA and flow cytometer, as described below. This experiment was replicated on five different pooled ejaculates collected from four bulls.

#### Computer-assisted sperm analysis

Fresh semen was initially evaluated following dilution (1:20) in tissue culture medium TCM-199 (37°C). Frozen semen samples were thawed and evaluated as such without further dilution in TCM-199. An aliquot (2.5 μl) of each semen sample was loaded in a pre-warmed (37°C) chamber slide (Leja Netherlands; 20 μM depth). At least 200 sperm were analyzed for total motility (% of all moving sperms), progressive motility (% of all sperms moving in straight line), average path velocity (VAP, μm/sec), curvilinear velocity (VCL, μm/sec) and straight-line velocity (VSL, μm/sec), as described earlier [33].

#### Flow cytometer analysis

Flow cytometer analysis was conducted to evaluate sperm plasma membrane (PM) and acrosomes (apical ridge, AR) integrities, as described previously [33]. After thawing, semen was diluted to 1x10^6^ sperm/ml with phosphate buffer saline (PBS) containing 0.5% w/v bovine serum albumin (BSA). Then, fluorescent dyes i.e. 1 μl fluorescein isothiocyanate-peanut agglutinin (FITC-PNA; Sigma chemicals, St. Louis, MO; stock 1 mg/ml in PBS) and 6.25 μl propidium iodide (PI; Invitrogen, Burlington, ON; stock 2.4 mM in water) were added to per ml semen and incubated for 10 min at 37°C. Sperm in each sample were fixed by adding 10 μl of 10% formaldehyde and analyzed within few hours. At least 10,000 sperm from each semen sample were analyzed using flow cytometer (Partec Cyflow Space, version 2.4 by Partec GmbH, Münster, Germany) equipped with 400 mW argon laser. FITC-PNA and PI were excited with 488 nm blue laser and fluorescent data were recorded with photo multiplier detectors FL-1 and FL-3, respectively, after gating sperm population with forward and side scatters. Four sperm populations were recorded on two dimensional PI/FITC-PNA dot plot [33]: sperm with intact plasma membrane and intact acrosomes (IPM-IAR; PI-/FITC-PNA-), sperm with intact plasma membrane and compromised acrosomes (IPM-CAR; PI-/FITC-PNA+), sperm with compromised plasma membrane and intact acrosomes (CPM-IAR; PI+/FITC-PNA-), and compromised plasma membrane and compromised acrosomes (CPM-CAR; PI+/FITC-PNA+).

### Electrophoresis of bull sperm frozen with and without egg yolk

Proteins were extracted from fresh sperm (control), and sperm frozen in CC2+TG7% and TEYG control extenders. Extenders CC2+TG7% and TEYG control were also run in electrophoresis, as controls. Fresh semen (100 μl) was centrifuged twice at 2000x g for 10 min in PBS to remove seminal plasma. The sperm pellet was resuspended in 100 μl extraction buffer [1% (w/v) sodium deoxycholate, 0.1% (w/v) sodium dodecyl sulphate and 1% (v/v) Triton-X in 1X PBS, pH 7.2] containing protease inhibitor cocktail (SigmaFast^™^ Cat # S8820; Sigma, St. Louis, MO), stored at -80°C and called as “Fresh sperm”. Semen straws frozen in CC2+TG7% and TEYG control extenders were thawed in a water bath at 37°C for 60 s. Egg yolk-free thawed semen (CC2+TG7%) was centrifuged twice at 500x g for 10 min in PBS. The sperm pellet was resuspended in 100 μl extraction buffer and called as “Frozen sperm-CC2+TG7%”. Egg yolk-containing thawed semen (TEYG control) was divided into two aliquots. In aliquot one, frozen-thawed semen (100 μl) was layered on 2.0 ml 45% Percoll in PBS and centrifuged at 2000x g for 10 min. The sperm pellet was resuspended in PBS and centrifuged at 1800x g for 5 min to remove Percoll particles. The sperm pellet was resuspended in 100 μl extraction buffer and called as “Frozen sperm-TEYG-PW”. In aliquot two, sperm were washed twice by centrifuging at 500x g for 10 min in PBS to remove egg yolk extender. The sperm pellet was resuspended in 100 μl extraction buffer and called as “Frozen sperm-TEYG-CW”. All sperm mixtures in extraction buffer were kept on ice for 30 min while mixing every 10 min with a pipette, sonicated for 10 min, centrifuged at 15,000x g for 15 min at 4°C and supernatant was used for electrophoresis. The protein concentration of each semen sample was measured with a Bio-Rad protein assay. Protein samples were mixed with 5X sample buffer, boiled for 5 min and cooled on ice. Total 15 μg protein from each fresh and frozen semen sample and TEYG control extender were loaded on 10% acrylamide gel. Since no protein was detected in CC+TG extender (2 mg/ml in TG extender), a volume equivalent to TEYG control extender was loaded on gel to confirm lack of protein in CC+TG extender. Electrophoresis was done at room temperature initially at 75 V for 15 minutes and then at 100 V until the bromophenol blue front line reached the bottom of the gel. Gels were stained with coomassie blue stain [0.1% (w/v) coomassie brilliant blue, 50% (v/v) methanol, 10% (v/v) acetic acid in water] on a shaking platform for 1 h. The extra stain was removed by destaining solution [10% (v/v) methanol and 40% (v/v) acetic acid in water] for 30 minutes on a shaking platform. The stained gels were scanned with Gel Doc^™^ EZ System (Bio-Rad Laboratories, Mississauga, ON, Canada) for protein profiling. This experiment was replicated three times using fresh and frozen-thawed sperm processed on different days.

### In vitro fertilizing ability of bull semen frozen with and without egg yolk egg yolk

Semen frozen with CC2+TG7% and TEYG control extenders were used to assess their in vitro fertilizing ability of bovine oocytes. *In vitro* maturation, fertilization and culture procedures were conducted as described previously [34]. Ovaries were collected from slaughtered cows and transported at 22°C to the Cryobiology Laboratory within 8 h. Immature cumulus oocyte complexes (COCs) were aspirated from 3–8 mm follicles. Under stereomicroscope, COCs containing more than three layers of cumulus cells and uniform cytoplasm were selected and washed (3x) in maturation medium TCM-199 supplemented with 5% (v/v) calf serum (CS), 5 μg/ml luteinizing hormone, 0.5 μg/ml follicle stimulating hormone and 0.05 μg/ml gentamicin). Groups of 20 COCs were incubated in 100 μl droplets of maturation medium, under mineral oil at 38.5°C, 5% CO_2_ in air and saturated humidity for 22 h. Frozen semen was thawed at 37°C for 60 s and washed through 2.0 ml Percoll gradient (45% and 90%). After washing, sperm were added to Brackett-Oliphant (BO) fertilization medium to final concentration 3x10^6^/ml. Groups of 20 COCs were incubated in 100 μl BO-sperm droplets at 38.5°C, 5% CO_2_ in air and saturated humidity. After 18 h of COC-sperm coincubation, cumulus cells and sperm attached to oocytes were mechanically removed via pipetting. Presumptive zygotes were washed (3x) in Charles Rosenkrans1+amino acids (CR1aa) *in vitro* culture (*IVC*) medium [35]. Twenty zygotes were transferred into 100 μl IVC droplets under mineral oil, and incubated at 38.5°C under 5% CO_2_, 90% N_2_, 5% O_2_ and saturated humidity. After 48 h incubation, the cleavage (2–8 cells) rate was recorded, and embryo culture continued in the same droplet. Subsequently, the blastocyst rate was determined on Day 9 (Day 0 = day of *IVF*). Both cleavage and blastocyst rates were calculated out of total number of oocytes fertilized. This experiment was replicated three times using fresh and frozen-thawed sperm processed on different days.

### Statistical analysis

Data were expressed as mean±SEM and analyzed using SAS® MIXED procedure (version 9.2, SAS institute Inc. Carry, NC). In first experiment, a 2x2 factorial design was used to observe the effects of CC and temperature of addition of glycerolated extender (TG). Later, data were pooled over temperature due to its non-significant effect on sperm characteristics and a completely randomized design was used to compare semen in different extenders. In second experiment, the effects of cholesterol-cyclodextrin and glycerol combinations were analyzed using a completely randomized design. Where P < 0.05, means were separated using Tukey’s test. Cleavage and blastocyst rates in TEYG control and CC+TG semen were analyzed using the Chi-square test.

## Results

### Cryopreservation of semen without egg yolk (exogenous protein)

**Fig 2:**
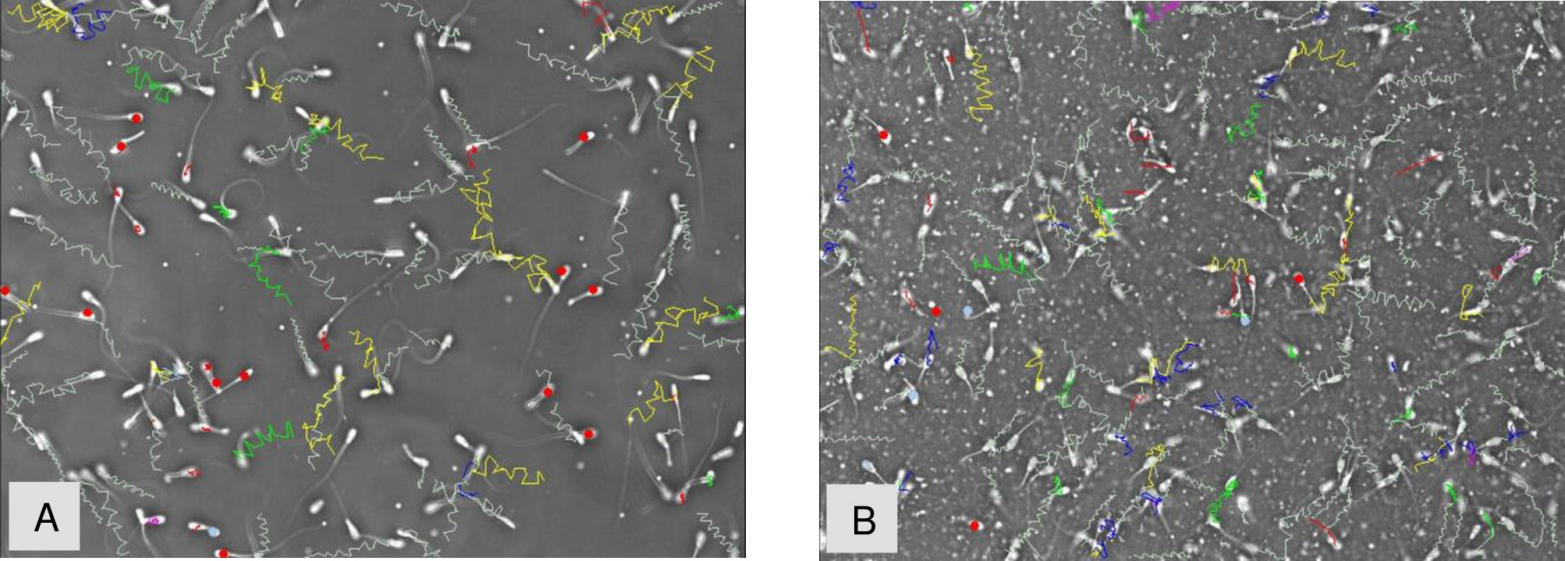
Post-thaw bull sperm diluted in CC2+TG7% (A) and TEYG control (B) extenders, under CASA microscope.

**Fig 3:**
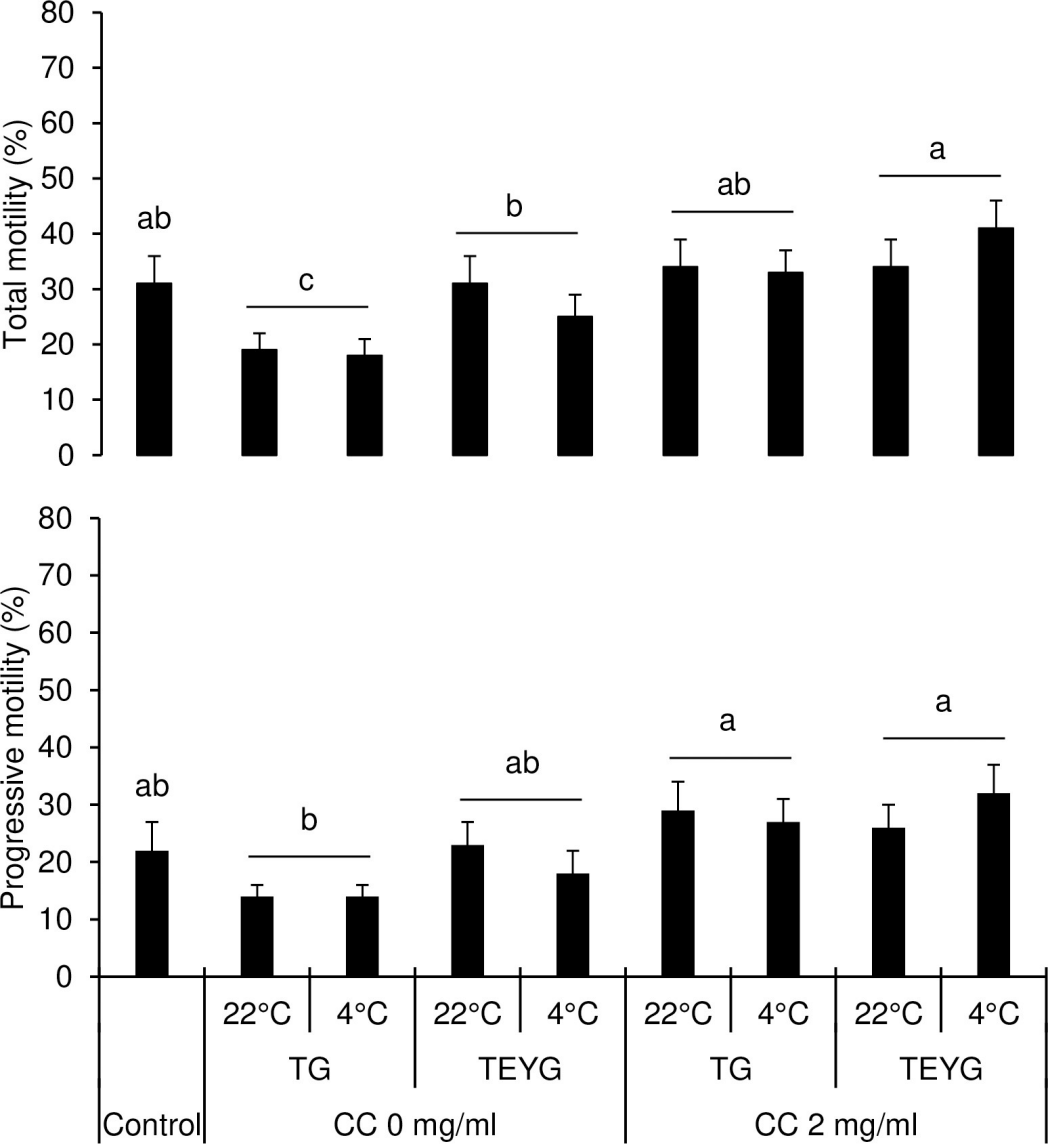
Effect of cholesterol-cyclodextrin complex, extender and temperature of glycerol addition on post-thaw sperm total motility and progressive motility. Each bar represents mean±SEM of five pooled ejaculates (replicates) from four bulls. Within a motility characteristic, bars with different letters (a-c) differ from each other (*p <* 0.05). Abbreviations: CC, cholesterol-cyclodextrin complex; EY, egg yolk; TG, tris-glycerol extender; TEYG, tris-egg yolk-glycerol extender.

**Fig 4:**
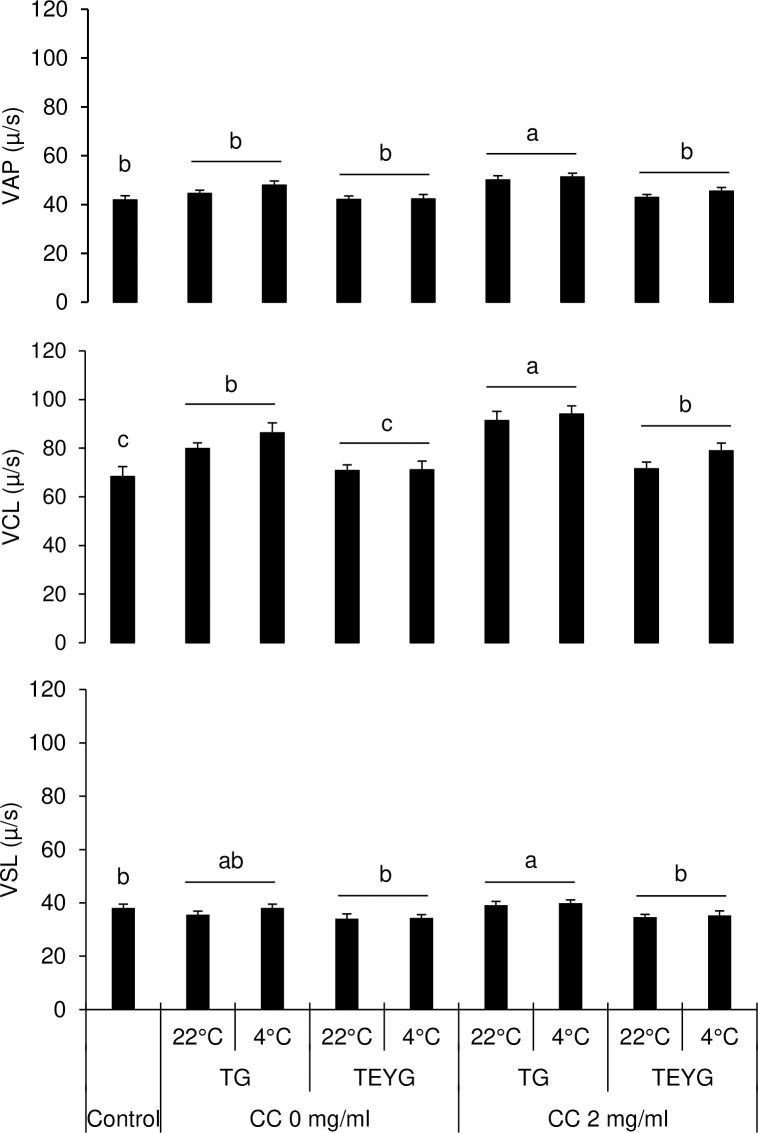
Effect of cholesterol-cyclodextrin complex, extender and temperature of glycerol addition on post-thaw sperm average path velocity (VAP), curvilinear velocity (VCL) and straight-line velocity (VSL). Each bar represents mean±SEM of five pooled ejaculates (replicates) from four bulls. Within a velocity characteristic, bars with different letters (a-c) differ from each other (*p <* 0.05). Abbreviations: CC, cholesterol-cyclodextrin complex; EY, egg yolk; TG, tris-glycerol extender; TEYG, tris-egg yolk-glycerol extender.

The background of post-thaw sperm diluted in CC+TG extender was more clear than in TEYG control extender, under CASA microscope (Fig 2). The effects of temperature of glycerol addition and extender x temperature of glycerol addition interaction on motion characteristics were not significant. Data on sperm motility and velocity characteristics in control and treatment groups are presented in Fig 3 and Fig 4. Sperm total and progressive motilities in TEYG control and CC-treated groups (with and without egg yolk) were similar (P > 0.05). Sperm total and progressive motilities were the lowest (P < 0.05) when both CC and egg yolk were absent in extender. Post-thaw sperm VAP, VCL and VSL were higher (P < 0.05) in CC2-+TG7% than in TEYG control extenders.

### Effect of cholesterol-cyclodextrin complex and glycerol combinations in extender on cryopreservation of semen without exogenous protein

**Fig 5:**
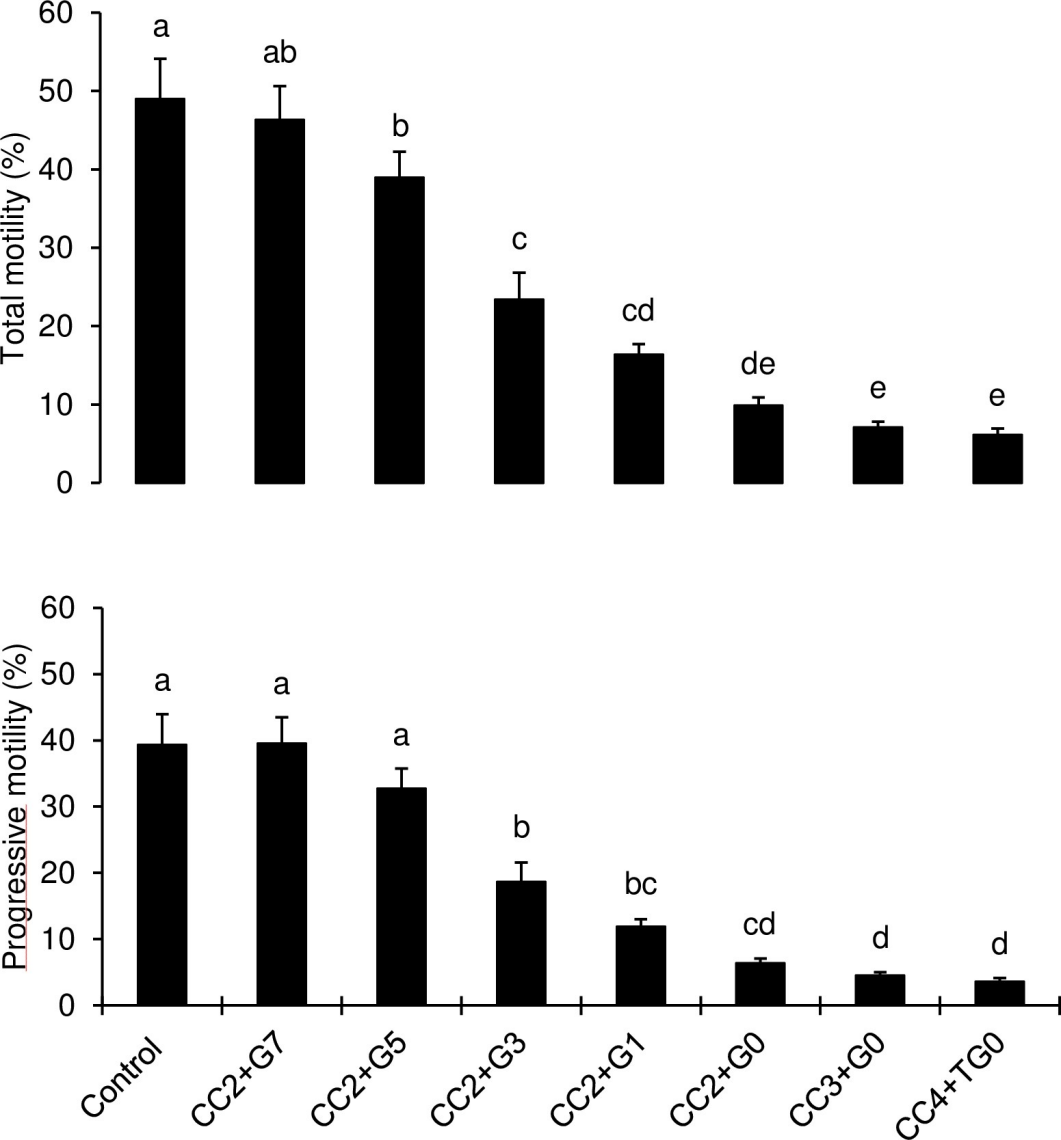
Effect of different concentrations of cholesterol-cyclodextrin complex and glycerol on post-thaw sperm total motility and progressive motility. Each bar represents mean±SEM of five pooled ejaculates (replicates) from four bulls. Bars with different letters (a-e) within a sperm motility characteristic differ from each other. Number (2, 3 or 4) following letters CC represents concentration of cholesterol-cyclodextrin complex (mg/ml). Number (7, 5, 3, 1 or 0) following letter G represents glycerol concentration in TG extender.

**Fig 6:**
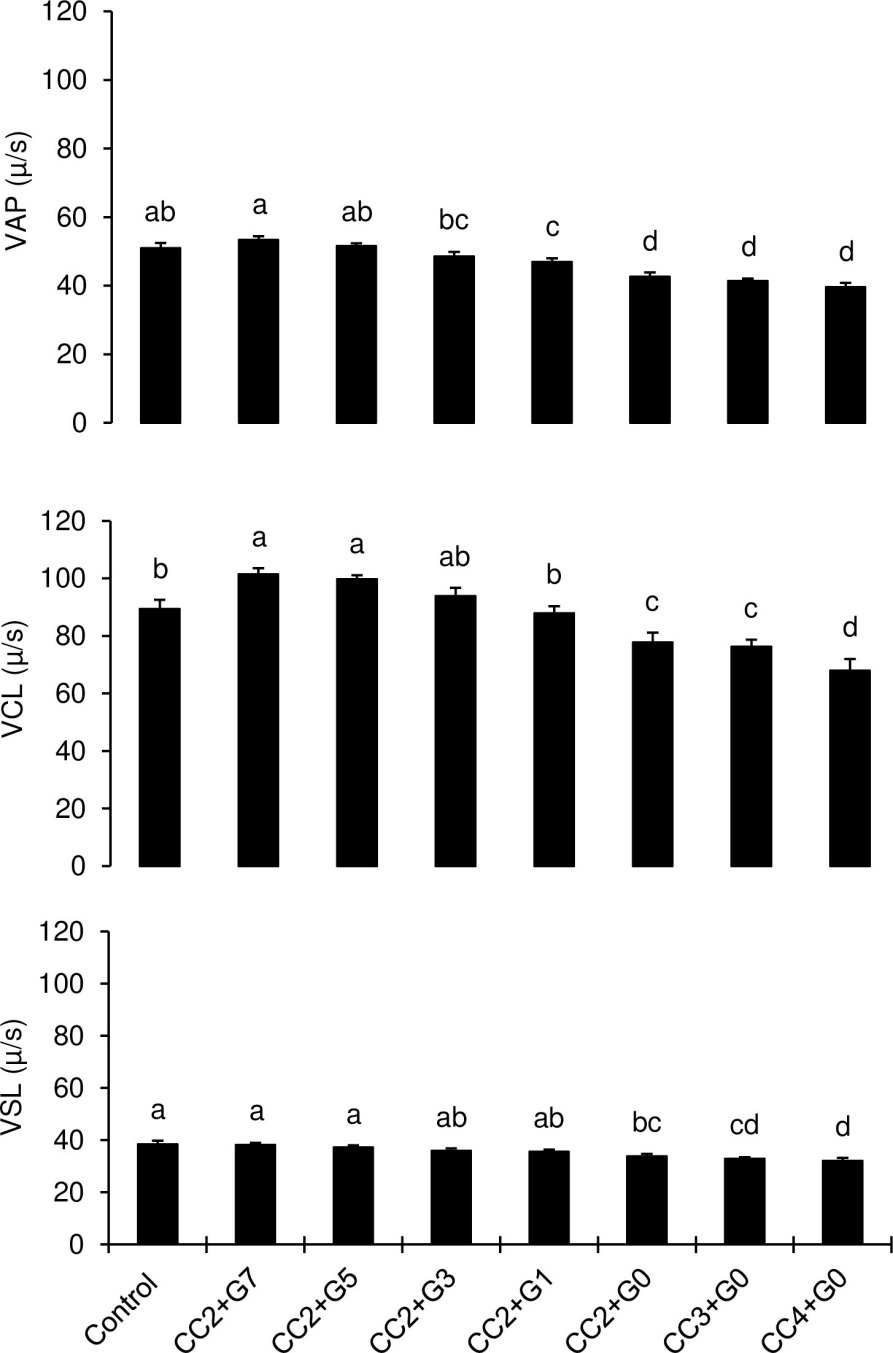
Effect of different concentrations of cholesterol-cyclodextrin complex and glycerol on post-thaw sperm average path velocity (VAP), curvilinear velocity (VCL) and straight-line velocity (VSL). Each bar represents mean±SEM of five pooled ejaculates (replicates) from four bulls. Bars with different letters (a-d) within a sperm motility characteristic differ from each other. Number (2, 3 or 4) following letters CC represents concentration of cholesterol-cyclodextrin complex (mg/ml). Numbers (7, 5, 3, 1 or 0) following letter G represent glycerol concentration in CC+TG extender.

Post-thaw motilities and velocities of bull sperm frozen in CC+TG extender containing ≤ 7% glycerol concentration are presented in Fig 5 and Fig 6 respectively. Post-thaw sperm total and progressive motilities declined as glycerol concentration decreased in TG extender. Sperm total and progressive motilities were higher (P < 0.05) in TEYG control and CC2+TG7% semen than other treatments possessing low glycerol concentration. Both sperm total and progressive motilities were the lowest (P < 0.05) in TG extender without glycerol (TG0%) in CC2, CC3 and CC4 treatment groups. Post-thaw sperm average path velocity was similar among TEYG control, CC2+TG7% and CC+TG5% (P > 0.05). Sperm curvilinear velocity was higher (P < 0.05) in CC2+TG7% and CC2+TG5% than in TEYG control semen. Sperm straight-line velocity was higher (P < 0.05) in CC+TG extenders than in glycerol-free (TG0%) extenders. The first significant decline in sperm VAP, VCL and VSL was observed when glycerol concentration in TG extender was reduced to 3%, 1% and 0% respectively.

Post-thaw sperm with intact plasma membrane and intact acrosomes (IPM-IAR), and sperm with compromised plasma membrane and intact acrosomes (CPM-IAR) populations were the highest in TEYG control semen but these populations decreased with a concomitant increase in sperm population having compromised plasma membrane and compromised acrosomes (CPM-CAR), as glycerol concentration decreased in TG extender (Table 1). Sperm population with intact plasma membrane and compromised acrosomes (IPM-CAR) was negligible (<3%). Like motilities, post-thaw sperm IPM-IAR were significantly low (P < 0.05) in extenders without glycerol i.e. CC2+TG0%, CC3+TG0% and CC4+TG0%.

**Table 1:** Post-thaw plasma membrane and acrosome integrities of bull sperm in TEYG control and CC+TG extenders containing lower than conventional glycerol concentration.

	IPM-IAR	IPM-CAR	CPM-IAR	CPM-CAR
TEYG control	40±3.7^a^	2±0.3^a^	20±1.0^a^	38±3.2^f^
CC2+TG7%	33±3.2^b^	1±0.3^a^	9±1.0^b^	57±3.7^e^
CC2+TG5%	33±3.4^b^	1±0.2^a^	10±1.3^b^	56±3.6^e^
CC2+TG3%	20±3.3^c^	1±0.2^a^	10±1.2^b^	69±3.3^d^
CC2+TG1%	12±1.8^d^	3±1.5^a^	12±1.7^b^	73±2.6^cd^
CC2+TG0%	4±0.4^e^	1±0.1^a^	10±1.3^b^	85±1.4^a^
CC3+TG0%	4±0.6^e^	1±0.2^a^	12±1.5^b^	83±2.1^ab^
CC4+TG0%	4±0.6^e^	3±1.8^a^	12±1.9^b^	77±4.2^bc^

TEYG control group represents bull sperm frozen in conventional TEYG extender containing egg yolk (20%, v/v) and glycerol (7%, v/v). In treatment groups, bull sperm were pre-exposed to cholesterol-cyclodextrin complex (CC; 2, 3 or 4 mg/ml) at 22°C for 15 min and then diluted in TG extenders containing different glycerol concentrations (G; 7, 5, 3, 1 or 0%, v/v) at 22°C. Data are presented as mean±SEM of five pooled ejaculates (replicates) from four bulls. Means with different superscripts^a-f^, within a column, differ from each other (P < 0.05).

Abbreviations: TEYG: tris-citric acid-egg yolk glycerol extender; CC: cholesterol-cyclodextrin complex; G: glycerol; IPM-IAR: intact plasma membrane and intact acrosomes; IPM-CAR: intact plasma membrane and compromised acrosomes; CPM-IAR: compromised plasma membrane and intact acrosomes; CPM-CAR: compromised plasma membrane and compromised acrosomes.

### Electrophoresis of bull sperm frozen with TEYG control and CC+TG extenders

**Fig 7:**
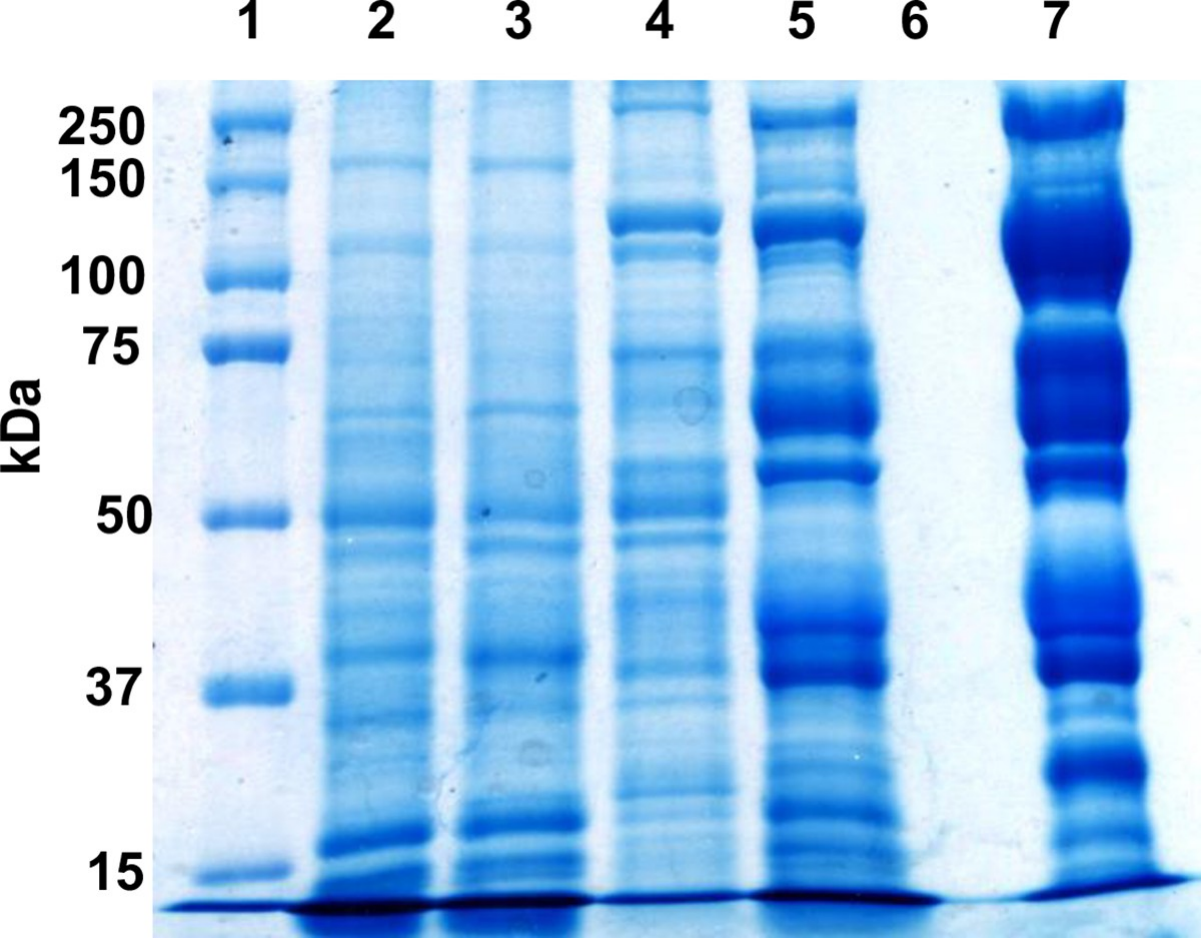
Representative SDS gel electrophoresis of proteins extracted from fresh and frozen-thawed bull sperm, cholesterol-cyclodextrin tris-glycerol (CC2+TG7%) extender and tris-egg yolk-glycerol (TEYG control) extender. Lane distribution: 1, Markers; 2, Fresh sperm; 3, Frozen sperm-CC2+TG7%; 4, Frozen sperm-TEYG-PW; 5, Frozen sperm-TEYG-CW; 6, CC2+TG7% extender per se; and 7, TEYG control extender per se. Lanes 2–5 and 7 contained 15 μg protein extract, lane 6 contained plain CC+TG extender (equivalent to the volume of TEYG extender used in lane 7).

Protein profiles from fresh and frozen sperm (diluted in CC+TG7% and TEYG control extenders), CC+TG extender and egg yolk extender alone are presented in Fig 7 and Table 2. Sperm protein profiles between fresh and frozen in CC2+TG7% were almost similar and 19 proteins (180, 113, 101, 92, 83, 73, 64, 60, 50, 47, 44, 42, 39, 33, 28, 27, 22, 18 and 14 kDa) were common between two semen types. Total 9 proteins (70, 58, 51, 41, 36, 31, 24, 20 and 14 kDa) were found common between TEYG control extender and sperm frozen in egg yolk (TEYG-PW and TEYG-CW).

**Table 2:** Protein profile (molecular weight [kDa] and relative band intensity %) of fresh and frozen-thawed bull sperm, and tris-egg yolk-glycerol extender.

Fresh sperm		Frozen sperm-CC2+TG7%		Frozen sperm-TEYG-PW		Frozen sperm-TEYG-CW		TEYG extender	
MW (KDa)	Band %	MW (KDa)	Band %	MW (KDa)	Band %	MW (KDa)	Band %	MW (KDa)	Band %
≥250	12.1	≥250	10.6	≥250	27.9	≥250	9.8	≥250	6.2
240	4.0	236	2.9	168	0.1	217	3.7	199	8.3
**180**	2.7	**180**	3.2	148	0.4	138	1.1	133	1.0
**113**	1.2	**111**	0.5	120	6.6	114	6.9	98	24.7
**101**	0.1	**98**	0.1	107	2.6	103	2.1	**70**	4.7
**92**	0.2	**91**	0.2	99	0.3	90	0.1	**58**	3.3
**83**	0.7	**84**	0.3	89	0.3	**71**	3.8	**51**	4.8
**73**	0.5	**73**	0.6	83	0.5	**61**	9.7	**41**	16.1
69	0.1	**64**	1.2	**71**	4.7	**54**	5.1	**36**	8.2
**64**	0.6	**60**	0.5	65	3.9	47	0.2	**31**	0.6
**60**	1.4	**50**	3.7	**55**	2.2	**40**	14.0	**24**	6.9
**50**	4.6	**47**	1.3	**50**	6.3	**37**	8.6	**20**	1.1
**47**	1.1	**44**	0.6	47	1.9	**33**	1.0	**14**	5.9
**44**	0.1	**42**	1.0	44	0.6	28	0.3	≤10	8.1
**42**	0.9	**39**	6.4	**41**	4.9	**24**	3.7		
**39**	1.8	**34**	1.4	**38**	3.0	**19**	1.9		
**33**	2.0	30	0.1	**34**	1.0	17	1.1		
**28**	0.4	**29**	0.1	29	0.4	**14**	11.9		
**27**	0.3	**28**	0.2	**24**	3.5	12	6.5		
**22**	6.7	**23**	7.6	**20**	2.0	≤10	8.5		
**18**	4.2	**18**	5.9	**14**	8.7				
**14**	41.8	**14**	37.3	≤10	18.0				
≤10	12.3	≤10	14.4						

Proteins were extracted from fresh sperm and sperm frozen in either CC2+TG7% or TEYG control extenders. After thawing, sperm frozen in egg yolk were either washed through Percoll gradient (Frozen sperm-TEYG-PW) or centrifuged twice (Frozen-TEYG-CW). Molecular weights and relative band densities were determined using Bio-Rad’s Gel Doc^™^ EZ System. Values in bold represent common proteins between fresh and Frozen sperm-CC+TG sperm. Values in bold and underlined represent common proteins between Frozen sperm-TEYG-PW, Frozen sperm-TEYG-CW and TEYG control extender per se. Protein bands between treatments with molecular weight difference ± 3 kDa were considered similar.

### In vitro fertilizing ability of bovine semen frozen with TEYG control and CC+TG extenders

Following in vitro fertilizing of bovine oocytes, semen frozen in TEYG control and CC2+TG7% extenders yielded similar cleavage and blastocyst rates (P > 0.05; Table 3).

**Table 3:** In vitro cleavage and blastocyst rates of bull semen frozen with TEYG control and CC2+TG7% extenders (Mean±SEM; N = three replicates).

Semen	No. of oocytes	Cleavage rate	Blastocyst rate
TEYG control	210	54±8.1	28±5.5
CC2+TG7%	202	54±6.3	28±5.5
	P-value	> 0.05	> 0.05

Abbreviations: TEYG: tris-egg yolk-glycerol extender; CC: cholesterol-cyclodextrin complex; TG: tris-glycerol.

## Discussion

This study reported successful cryopreservation of bull semen without adding any exogenous protein (animal or plant origin) in semen extender. Treatment of bull sperm with CC complex completely eliminated the need of egg yolk from conventional semen cryopreservation procedure. However, CC complex could not replace glycerol which remained an essential ingredient of extender to protect sperm during the sub-zero phase of cryopreservation. Bull sperm cryopreserved with and without egg yolk demonstrated similar post-thaw motility and in vitro fertilizing ability. As a proof of concept, sperm frozen in CC+TG extender (without egg yolk) exhibited similar protein profile as the fresh sperm; whereas, proteins in egg yolk TEYG control extender bound tightly with bull sperm plasma membrane.

This study demonstrated that post-thaw total and progressive motilities were similar in CC+TG (egg yolk-free) extender and in conventional TEYG control extender. Regardless of the presence or absence of egg yolk, treatment of sperm with CC complex improved their post-thaw motility as compared without CC (control). It is anticipated that lower post-thaw velocities of sperm in TEYG control extender than in CC+TG extender may be due to the high density of egg yolk content. These observations indicated that bull sperm can be successfully frozen without egg yolk if they are treated with CC complex before dilution in egg yolk-free glycerolated extender. It will eliminate the biosecurity risk associated with animal proteins in extender. Cholesterol stabilizes the sperm plasma membrane [36]. During initial cooling phase from room temperature to 0°C (supra-zero phase), plasma membranes undergo thermotropic phase transition which ultimately destabilizes the plasm membrane organization. Cholesterol protects sperm during the supra-zero phase and modulates thermotropic membrane phase behavior [37]. High cholesterol:phospholipid ratio reduces membrane disorganization caused by phase transition [38]. Egg yolk, a common ingredient in extender, contributes its lipid proteins, cholesterol and antioxidant moieties to the sperm membrane [7]. Low density lipoprotein fraction present in egg yolk protects bull sperm during initial cooling phase [2,3]. Egg yolk particulates in extender overestimate sperm concentration in computer-assisted sperm analysis and interfere with motility evaluation [13,39]. Under CASA examination, the background of semen diluted was more clear in CC+TG than egg yolk diluted semen which made semen evaluation easy.

Total and progressive sperm motilities were the lowest when semen was frozen without CC complex treatment and egg yolk in extender. This indicated that glycerol alone in extender could not protect sperm during initial cooling (supra-zero) phase. Glycerol is known cryoprotectant which protects sperm below sub-zero temperature. Bull sperm need CC complex treatment and/or egg yolk-based extender to survive the initial cooling phase from room to 0°C. In this study, a true protective effect of CC complex during initial cooling phase was determined by adding the glycerolated (TG) extender at 22 or 4°C. Previously, it was reported that the addition of glycerolated extender at 5°C provided better protection to bovine sperm during freezing compared to glycerol addition at >5°C [40]. In this study, the lack of difference in total and progressive motilities due to temperature of glycerol addition (22 vs. 4°C) confirmed a true protective effect of CC complex alone during the initial cooling phase. In subsequent experiments, the tris-glycerol (TG) extender was added in semen at 22°C, for the sake of convenience.

The present study suggested that post-thaw total and progressive motilities and velocities were dependent upon glycerol concentration in TG extender. Sperm motion characteristics declined with decrease in glycerol concentration in TG extender. Even higher concentration of CC complex (3 and 4 mg/ml) without glycerol could yield only <10% post-thaw sperm motility and IPM-IAR. In contrast, using HPβCD-C complex, the glycerol concentration was reduced from routine 3% to 1% for cryopreservation of equine sperm [27]. This difference could be attributed to difference in sperm plasma membrane composition of two species, and chemical nature of cyclodextrins used in two studies. There was a only 7% decline in sperm with IPM-IAR in CC2+TG7% and CC2+TG5% extenders as compared with TEYG control extender. Bull plasma membrane and acrosomes seem sensitive to glycerol concentration in TG extender. Glycerol activated various caspases which led human sperm to undergo programmed cell death [41]. This study recommended that treatment of sperm with 2 mg/ml CC complex and 5% to 7% glycerol in TG extender is suitable combination for freezing of bull semen without egg yolk.

Post-thaw protein profile of sperm frozen in CC+TG extender was similar to protein profile of fresh sperm. It is suggested that CC+TG extender is suitable extender to study changes in sperm proteins due to cryopreservation per se without any modifications by exogenous proteins (e.g. egg yolk). This approach will provide an opportunity to identify and isolate freeze-tolerant membrane proteins in mammalian sperm and to improve the freezing ability of semen from poor freezer species. This study showed that egg yolk proteins bound tightly with sperm membrane and the majority of proteins could not be removed completely even after Percoll washing and double centrifugation. It makes difficult to study changes in plasma membrane proteins of sperm diluted in egg yolk extender.

In vitro fertilization of bovine oocytes with semen frozen in TEYG control or CC+TG extenders yielded similar cleavage and blastocyst rates. It indicated semen frozen without egg yolk has the capability to fertilize cows’ oocytes in vitro. In vivo fertility trials of semen frozen without egg yolk are in progress and will be reported shortly.

In conclusion, bull semen can be frozen successfully without egg yolk by treating sperm with CC complex alone. It appears that CC complex protected bull sperm during the initial (supra-zero) cooling phase. Glycerol, regardless of its addition at room or refrigerated temperature, is still required to protect bull sperm during the deep (sub-zero) freezing phase. Electrophoresis data revealed that protein profiles of plasma membrane of fresh sperm and frozen sperm TG (without egg yolk) are similar. In contrast, egg yolk proteins bind and modify sperm plasma membrane. Both, semen frozen with and without egg yolk has similar fertilization potential in vitro.
